# High-Order Aberrations in Cataract Surgery: Current Status and Future Perspectives: A Scoping Review

**DOI:** 10.3390/medicina62030512

**Published:** 2026-03-10

**Authors:** Andreea Alexandra Mihaela Musat, Calin Petru Tataru, Gabriela Cornelia Musat, Vanda Roxana Nimigean, Mihai Alexandru Preda, Ovidiu Musat

**Affiliations:** 1 Doctoral School, Carol Davila University of Medicine and Pharmacy, 050474 Bucharest, Romania; andreea-alexandra.musat@rez.umfcd.ro; 2 Ophthalmology Department, Central Emergency University Military Hospital, 010825 Bucharest, Romania; calin.tataru@umfcd.ro (C.P.T.); ovidiu.musat@umfcd.ro (O.M.); 3 Ophthalmology Department, Carol Davila University of Medicine and Pharmacy, 050474 Bucharest, Romania; 4 ENT Department, Saint Mary Clinical Hospital, 011172 Bucharest, Romania; mihai.preda@umfcd.ro; 5 ENT Department, Carol Davila University of Medicine and Pharmacy, 050474 Bucharest, Romania; 6 Department of Oral Rehabilitation, Faculty of Dentistry, Carol Davila University of Medicine and Pharmacy, 4-6 Eforie Street, 050037 Bucharest, Romania

**Keywords:** cataract surgery, higher-order aberrations, intraocular lenses, wavefront aberrometry, refractive outcomes

## Abstract

*Background and Objectives*: Due to the evolution of cataract surgery into a refractive procedure in which optimizing visual quality extends beyond achieving good visual acuity, high-order aberrations have been increasingly recognized as important contributors to postoperative visual performance. This scoping review aims to map and synthesize the available evidence on higher-order aberrations in the context of cataract surgery, with a focus on the surgical techniques, intraocular lens (IOL) design, measurement factors, and their association with the visual outcomes. *Materials and Methods*: A scoping review was conducted in accordance with the PRISMA-ScR guidelines. A search of electronic databases was performed using a predefined Boolean strategy, complemented by a manual screening of reference lists and independent searches. Studies evaluating higher-order aberrations (HOAs) before and/or after cataract surgery were included. Data were charted descriptively, and findings were synthesized narratively. *Results:* A total of 94 studies were included. The evidence shows that differences in HOA profiles are based on the surgical techniques, IOL designs (monofocal, multifocal, toric, and extended depth-of-focus), and measurement devices. Increased HOAs were frequently associated with reduced contrast sensitivity, especially under mesopic conditions. Tear-film instability and pupil size were additional factors contributing to dynamic changes in wavefront aberrations. Considerable methodological heterogeneity was observed across studies. *Conclusions*: The current body of evidence suggests a strong connection between corneal characteristics, IOL design, surgical techniques, patient-specific factors, and postoperative visual quality. HOAs play an important but not an exclusive role. Future research should focus on standardized measurement approaches, population-specific optical considerations, and personalized strategies to optimize visual quality after cataract surgery.

## 1. Introduction

### 1.1. Context

In recent years, cataract surgery has changed its purpose from ocular function restoration to maximizing visual performance [1]. Being recognized as one of the most successful and cost-effective surgical interventions, it is performed millions of times annually worldwide [2]. Continuous technological advances have made cataract surgery the safest and most predictable ophthalmic procedure, but the ongoing pursuit of excellence fuels innovation and progress aimed at enhancing optical quality and patient-perceived visual outcomes [3]. Traditionally, cataract surgery focused on the removal of the opacified lens and the implantation of an intraocular lens (IOL). However, the modern surgical practice focuses on a refractive and individualized approach, taking into account factors such as the corneal shape, astigmatism, and higher-order aberrations (HOAs) [1].

### 1.2. Higher-Order Aberrations (HOAs) and Vision Quality

The human eye functions as a sophisticated system and is composed of multiple structures, each contributing to the overall image quality. Imperfections, known as optical aberrations, can affect the clarity of the image and limit visual performance [4]. The air-cornea interface has the greatest effect on the total refraction because of the substantial refractive index difference between the air and the corneal tissue and is responsible for approximately 90% of the eye’s HOAs [5,6]. Analysis revealed that the distribution of the posterior–anterior corneal radius ratio (B/F ratio) in individuals with no corneal abnormalities negatively correlated with the corneal thickness and aberrations while demonstrating a positive association with posterior corneal geometry, axial length, and anterior corneal curvature [7]. In young individuals, the crystalline lens is able to effectively compensate for the cornea’s inherent positive spherical aberration. Although the cornea remains relatively stable throughout life, the optical properties of the crystalline lens change with the aging process, losing its compensatory function as its spherical aberration shifts towards positive values. This age-related change results in an overall increase in the eye’s total positive spherical aberration [8].

**Figure 1:**
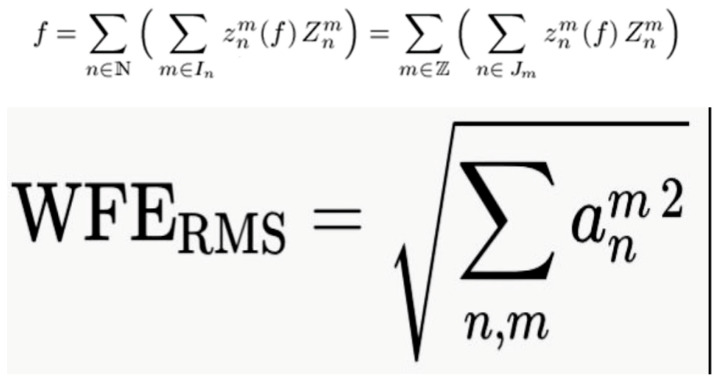
Wavefront error decomposed into Zernike polynomials. The total wavefront aberrations are equivalent to the sum of all non-zero root mean square (RMS) coefficients of the corresponding Zernike modes. Mathematically, the RMS value of the total wavefront is calculated as the square root of the sum of the squares of all individual coefficients; *m* and *n* are natural numbers representing the order of the Zernike polynomials. When the polynomials are orthonormal, the wavefront error can be simplified to the root mean square of the Zernike coefficients.

Cataract surgery may modify corneal HOAs either directly, by the structural changes induced in the corneal tissue itself, or indirectly, through the optical changes following IOL implantation. HOAs are mathematically represented by the Zernike polynomials, and the most clinically significant are coma, trefoil, and spherical aberration [9]. Zernike polynomials constitute a complete set of orthogonal functions defined over the unit circle, providing a convenient means of decomposing optical wavefronts into aberration components, such as astigmatism, coma, and spherical aberration. Their well-established relationship with classical aberration enables the characterization and quantification of optical imperfections [10]. Zernike polynomials can be either expressed using a single (Z_j_) or a double-index notation (Z_n_^m^). While the single-index notation is simple, the double-index notation provides more detailed information within a single term. In this system, the term denotes the aberration order, while the term m represents the spatial frequency of the wavefront aberration. In Figure 1, we decomposed the wavefront error into Zernike polynomials.

Because the interpretation of the Zernike coefficients can be difficult, the root mean square (RMS) metric is commonly used to assess ocular aberrations. RMS represents the deviation of the wavefront from its mean value and is calculated as the square root of the wavefront variance. It is expressed as a single numeric value, and it provides an intuitive and practical measure of the magnitude of the wavefront aberrations, facilitating clinical interpretation [11].

These phenomena can produce symptoms in patients such as glare, halos, and decreased contrast sensitivity, which may be present even when standard measures of visual acuity indicate excellent postoperative vision [1]. Until recently, clinicians were able to correct only low-order aberrations (LOAs), such as myopia, hypermetropia, and astigmatism. HOAs, which correspond to wavefront modes of the third radial order (n ≥ 3) and above, remained largely uncorrectable. Nowadays, technological advances have enabled a more refined approach in assessing and managing HOAs, extending beyond the correction of LOAs [6].

### 1.3. Rationale for the Review

Although modern cataract surgery achieves excellent visual acuity, HOAs can still limit the postoperative outcome. This review seeks to consolidate current knowledge on the influence of HOAs in the context of cataract surgery and to identify trends, gaps, and opportunities for future research and clinical practice.

### 1.4. Objectives

The purpose of this scoping review is to comprehensively map the available literature regarding HOAs in the context of cataract surgery. This review will categorize and identify evidence on (1) the prevalence and sources of HOAs, (2) methods and technologies used to measure these aberrations, (3) their impact on the visual postoperative outcomes, and (4) potential strategies to minimize HOAs through surgical planning, IOL selection, and optical correction. By consolidating current knowledge, this review aims to clarify the clinical relevance of HOAs and to support evidence-based approaches to optimize visual outcomes in modern cataract surgery.

## 2. Methods

### Protocol and Reporting Framework

This scoping review followed Arksey & O’Malley [12] and the Joanna Briggs Institute (JBI) guidelines, reported according to the Preferred Reporting Items for Systematic reviews and Meta-Analyses extension for Scoping Reviews (PRISMA-ScR) [13]. The review protocol was developed a priori and registered on the Open Science Framework Databases (OSF; Registration DOI 10.17605/OSF.IO/VYXNJ). A comprehensive literature search was performed in the PubMed/MEDLINE, Scopus, and the Cochrane Library from 2000 to 2025. The search strategy was based on predefined free-text terms related to cataract surgery, IOLs, and higher-order aberrations combined using Boolean operators (AND, OR). A structured Boolean search strategy was developed and applied in PubMed as the primary database: (“cataract surgery”) AND (“high order aberrations”) AND (“IOL”). The database search yielded a total of 108 publications after the application of predefined limits. No duplicates were found. Reference lists of included studies and relevant reviews were also screened to identify additional publications. For the remaining databases, searches were conducted using relevant keywords related to cataract surgery, IOLs, and higher-order aberrations. These searches were adapted to the interface and indexing structure of each database. The main keywords used in the additional databases included: “cataract surgery”, “intraocular lens”, “IOL”, “higher-order aberrations”, and “wavefront aberrations”. These searches were performed to identify supplementary literature and ensure broader coverage. Manual screening of reference lists of eligible articles was also performed in order to identify additional relevant studies. No restriction was applied regarding study design. Only studies published in the English language were considered eligible.

Studies were included if they met the following criteria: patients undergoing cataract surgery, intraocular lens implantation, assessment of higher-order aberrations using wavefront aberrometry or related optical measurements, randomized controlled trials, prospective or retrospective observational studies, and relevant reviews providing structural analysis of HOA outcomes. Studies were excluded if they involved animal subjects, did not include cataract surgery, did not report HOA measurements, or were not available in the English language.

Randomized controlled trials, cohort, cross-sectional, and case-series designs were included. Two reviewers independently screened the titles and abstracts, assessed full texts for eligibility, and resolved discrepancies by discussion and consensus. Data were charted using a standardized extraction form capturing study characteristics, surgical techniques, IOL type, HOA measurement method, and visual outcomes. Extracted data were summarized descriptively and thematically without quantitative pooling, consistent with the exploratory nature of scoping reviews. In Table 1, we present a list of inclusion and exclusion criteria of the studies included.

As this review was conducted as a scoping review, the primary objective was to map the available evidence rather than perform a formal quality-based exclusion. However, study design variability and potential sources of bias were considered during data interpretation. These included small sample sizes, short follow-up duration, heterogeneity in measurement devices, and differences in patient selection criteria.

Given the substantial heterogeneity across studies, a quantitative meta-analysis was not performed. Instead, a more narrative, qualitative approach was taken, structured around domains including surgical techniques, IOL design, measurement variability, and functional visual outcomes.

## 3. Results

### 3.1. Study Selection and Characteristics

**Figure 2:**
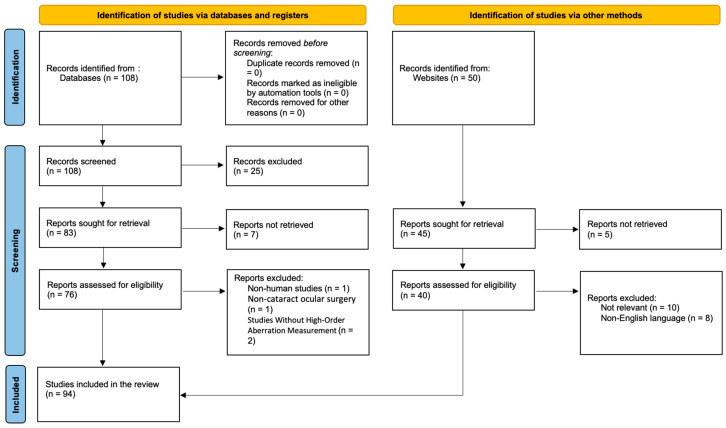
Flowchart of the literature review. The identification process followed two parallel pathways in accordance with PRISMA-ScR guidelines: (1) database searching and (2) additional sources (websites and reference screening). After screening and eligibility assessment, 72 studies were included from database searches and 22 from other sources, resulting in a final total of 94 studies included in the review.

Based on our database search, we identified a total of 108 publications. No duplicate records were found, and no records were excluded by automation tools or for other reasons prior to screening. All 108 records were screened by title and abstract, resulting in the exclusion of 25 records. Following screening, 83 reports were sought for full-text retrieval, of which 7 publications could not be retrieved. Consequently, 76 full-text articles were assessed for eligibility. Of these, 4 reports were excluded, including non-human studies (n = 1) and studies that did not include high-order aberration measurements (n = 2). Additionally, 50 records were identified through other methods, including website searches. From these, 45 publications were sought for retrieval, with 5 reports not retrieved, resulting in 40 reports assessed for eligibility. Among these, 18 reports were excluded, primarily because they were not relevant to the review in question (n = 8) or were published in a non-English language (n = 8). Overall, 94 studies met the inclusion criteria and were included in the final scoping review. In Figure 2, we present the flowchart of the literature review.

### 3.2. Surgical Techniques and HOAs

Visual function in eyes with IOLs is significantly associated with ocular, internal, and corneal HOAs [14]. The included studies evaluated the impact of cataract surgery-related factors, IOL design, and preoperative characteristics on HOAs and postoperative outcomes. Comparisons between different surgical techniques, including femtosecond laser-assisted cataract surgery and conventional phacoemulsification, revealed variability in corneal and total ocular HOAs.

### 3.3. IOL Design and Optical Characteristics

Studies comparing monofocal, multifocal, and extended depth-of-focus (EDOF) IOLs reported distinct optical characteristics. While multifocal and EDOF IOLs were associated with higher levels of patient satisfaction and spectacle independence, they were also associated with increased reports of photic phenomena and specific aberration patterns. Increased spherical aberration and coma were associated with reduced contrast sensitivity, particularly under mesopic or low-contrast conditions.

### 3.4. Measurement Variability

Aberrometry-based assessments demonstrated variability between measurement devices. In pseudophakic eyes, corneal aberrations were reported to reflect total optical quality better than in phakic eyes, although inconsistent correlations with total wavefront were found.

### 3.5. Clinical Correlations

In addition to surgical and optical factors, patient-related variables were reported to influence wavefront aberrations, such as tear-film instability and pupil size.

## 4. Discussion

This review highlights the measurable impact cataract surgery has on HOAs and the postoperative influence on visual acuity and the quality of life. Across included studies, it was concluded that the IOL design, the surgical technique, and the preoperative corneal profile significantly impacted the changes in total HOAs [1,15,16,17,18]. Particularly in patients with high visual demands, minimizing surgically induced HOAs may be essential to optimize the visual outcomes, especially because HOAs reduce the quality of the retinal image and decrease contrast sensitivity [19]. This is very important, especially in the context of refractive lens exchange, where patients typically have minimal visual disturbances and high expectations. A significant relationship between fourth-order wavefront aberrations and mesopic contrast sensitivity was observed in eyes implanted with spherical IOLs [20]. However, there are contradicting opinions in the literature regarding whether the reduction in HOAs following cataract surgery may be related to the replacement of the cataractous lens with an IOL rather than changes in the corneal architecture [1,21]. Although generally the contribution of the crystalline lens is not a parameter routinely evaluated in clinical practice, it has a great impact on the total HOAs of the eye. Several factors might explain this, including limited access to technologies capable of distinguishing corneal from lenticular HOAs and the lack of perceived necessity of such measurements. However, it is known that lenticular HOA values increase as the cataract progression occurs [22]. This suggests that a transparent crystalline lens is typically associated with low internal HOAs and therefore good visual acuity [15].

Additionally, several other factors, such as tear-film instability, might contribute to dynamic changes in wavefront aberrations [23]. Preoperative treatment of dry eye disease might contribute to better postoperative vision quality and patient satisfaction [24]. Associations have been demonstrated between pupil size and both increasing age and positive refractive errors. These correlations are relevant especially in the context of older individuals undergoing photoablative corneal refractive surgery and multifocal IOL implantation. Therefore, pupil size is a critical factor in evaluating and predicting outcomes following cataract extraction and IOL implantation [25]. Larger pupils are typically associated with an increase in HOAs and may influence postoperative visual quality [26,27,28,29,30,31]. In patients with multifocal IOLs, larger pupils have also been associated with more frequent symptoms of glare, halos, and decreased contrast sensitivity [29,30,31]. Conversely, they may provide a better uncorrected near visual acuity compared to smaller pupils, as larger pupils allow the light to enter through multiple foci of the multifocal lens, resulting in superimposed retinal images from different focal points [32,33]. The relationship between HOAs and refractive errors remains inconsistent in the literature [34]. Some investigations have reported no statistically significant correlation between HOAs and the type or the amount of refractive error, while others have found a strong association, especially with myopia [35,36,37,38,39,40].

IOL positioning plays a critical role in the postoperative visual quality. The magnitude of the IOL misalignment, including decentration and tilt, impacts the final outcome, highlighting the importance of precise IOL placement to ensure optimal visual outcomes [41,42]. Moreover, there is evidence suggesting that the implantation of capsular tension rings results in a reduction in HOAs, likely due to improved centration and stability of the IOL [43]. In addition, the IOL type has a significant impact. By incorporating one or two aspheric surfaces, aspheric IOLs are theoretically designed to neutralize the cornea’s inherent positive spherical aberration. This design aims to compensate for the total positive aberration typically observed in the aging eye [44]. When compared with spherical IOLs, aspherical IOLs have been shown to significantly reduce HOAs and improve contrast sensitivity [45,46,47,48,49,50,51]. Additionally, the data indicate that the current aspheric IOLs available might generate a level of negative spherical aberration that is not universally optimal, suggesting possible racial or ethnic variations and underscoring the need for further population-specific studies [52]. Many studies comparing different types of aspheric IOLs have been conducted [53,54,55,56,57]. Based on our observations, although HOAs showed some variability between groups, the final outcomes regarding patient satisfaction and visual acuity did not differ substantially. This suggests that higher-order aberrations are not the only factor influencing the overall postoperative subjective quality of vision. Remarkably, although sulcus-fixated IOLs exhibited levels of tilt and decentration beyond the tolerable limits for aspheric IOLs, spherical IOLs implanted in sulcus were found to attenuate corneal wavefront aberration in cases of capsular bag defects during cataract surgery [58].

Differences have been reported between monofocal, multifocal, and extended-depth of focus (EDOF) IOLs in terms of optical quality and visual performance. Traditionally, monofocal IOLs have been associated with lower levels of HOAs [59,60]. However, recent evidence suggests that multifocal IOLs can provide excellent visual outcomes and spectacle independence without significantly increasing wavefront aberrations. Despite these advantages, certain side effects, such as straylight, have been observed [61]. While some studies have suggested that reduced contrast sensitivity in patients with multifocal IOLs has been partially attributed to increased spherical aberration, other findings indicate that these implants are not associated with a statistically significant loss of contrast sensitivity [62,63,64,65]. Although the recovery time was longer, patient satisfaction has been reported to be higher in patients with multifocal IOLs than monofocal IOLs, largely attributed to their ability to perform near tasks without the need of corrective spectacles. Overall, most patients considered the optical disturbances to be minor in comparison to the visual functionality and independence [66]. Enhanced monofocal IOLs (also called “monofocal plus”) have been introduced to slightly extend the depth of focus while maintaining a predominantly monofocal optical profile. These lenses aim to provide a better intermediate vision with a minimal photic phenomena or HOA induction.

The pure extended depth of focus (EDOF) IOLs have an increased spherical aberration in order to elongate the focal range without introducing the concept of multifocality. By increasing certain ocular aberrations, these IOLs create a controlled amount of blur, enabling functional vision at various distances [67]. Although HOAs generally degrade the quality of the retinal image, evidence suggests that certain aberrations, such as spherical aberration, coma, and secondary astigmatism, can enhance depth of focus [68]. This optical principle forms the basis for the extended depth of focus achieved with EDOF IOLs. On the other hand, near visual performance remains limited with most EDOF IOLs [69]. While some studies have reported satisfactory near vision, the findings are inconsistent and remain a topic of debate [70]. Neuroadaptation plays a critical role in patients with multifocal or EDOF IOLs. It is a gradual process influenced by individual variability, and a sudden increase in aberrations may not always be well tolerated [71].

Regarding photic phenomena, the evidence is limited, but some studies suggest that EDOF IOLs are associated with lower incidence and intensity of glare and halos compared to multifocal IOLs [72,73].

EDOF IOLs offer a promising alternative to multifocal IOLs, with studies demonstrating high patient satisfaction with good optical quality and minimized visual disturbances [74,75].

Regarding toric IOLs, cases in which the postoperative residual astigmatism was higher than anticipated were associated with higher preoperative corneal aberrations, particularly vertical coma [76]. Some evidence shows that no correlations have been made between corneal, intraocular, and total higher-order aberrations between monofocal toric and non-toric IOLs [77]. In addition, eyes with toric IOLs and those with high preexisting astigmatism exhibited higher postoperative ocular and corneal HOAs, leading to decreased photopic low contrast and mesopic visual acuities compared to eyes with non-toric IOLs and low preexisting astigmatism [78].

Contrast sensitivity represents a functional marker of postoperative optical quality and may indicate subtle visual acuity degradation not captured by standard high-contrast visual acuity testing. Variability in contrast sensitivity across studies may reflect differences in IOL design, pupil size, lighting conditions, and measurement methodologies.

Evidence shows that cataract surgery performed with incisions sized 2.2 mm or smaller produces only minimal changes in the total corneal aberrations. These changes are more significant in eyes with preexisting astigmatism, might be primarily related to irregular surgically induced astigmatism, and seem to be independent of whether a toric or non-toric IOL is used [79]. On the other hand, clear corneal incision (CCI)-related parameters, such as incision length, angle, distance from the incision to the corneal center, and corneal thickness at the incision site, can influence the postoperative HOAs, increasing them at 1 month postoperatively. Although these effects seem to diminish over time, they could persist until 6 months postoperatively [80].

Regarding surgical incision, the femtosecond laser allows for precise control over the incision site, architecture, and size [81]. Femtosecond laser-assisted clear corneal incisions are highly reproducible, stable, self-sealing, and have excellent wound geometry, having the potential of reducing the mechanical stress during surgery [82]. Surgically induced astigmatism (SIA) has been reported to be smaller in femtolaser-assisted CCIs than in manual CCI, but no statistically significant difference was found. Additionally, no statistically significant differences in corneal HOAs were observed between femtosecond-assisted cataract surgery (FLACS) and manual CCI at any given point in time (preoperatively or postoperatively). However, a statistically significant difference regarding horizontal coma was detected, with the FLACS group showing lower values [83]. In addition, evidence shows that clear corneal tunnel phacoemulsification with IOL implantation guided by corneal topography might be more effective in terms of reducing the preexisting astigmatism and inducing fewer HOAs compared to the traditional temporal clear corneal tunnel phacoemulsification, and therefore achieving superior visual acuity [84].

Aberrometry is a non-invasive diagnostic method for evaluating imperfections in the eyes’ optical system. It is used to detect and quantitatively assess the wavefront errors of the visual system, providing valuable insight into both lower and higher order aberrations. Aberrometry techniques can be divided into objective and subjective approaches:

### 4.1. Objective Aberrometry

Objective methods measure optical aberrations without relying on the patient’s visual response. These methods are classified into 3 categories:

- Outgoing methods—the wavefront is analyzed as it exits the pupil; for example, the Hartmann-Shack wavefront sensor.
- Ingoing methods—analyzes how the light entering the pupil travels through ocular media to form a retinal image; for example, the Tserning aberroscope, cross-cylinder aberroscope, and ray-tracing techniques.
- Ingoing feedback methods: the patient’s feedback is used in a physiological task for the assessment of the wavefront aberrations; for example, spatially resolved refractometer.

### 4.2. Subjective Aberrometry

Subjective techniques depend on the patient’s visual perception to assess optical quality and aberrations, for example, the Scheiner-Smirnov aberrometer [11].

The Hartmann-Shack system remains the most employed in clinical settings [85,86]. There have been reports regarding differences in measurements between different aberrometers in terms of internal optical aberrations, regardless of pupil size. These discrepancies suggest that, even in healthy eyes, variations in device design, optical principles, and calculation algorithms may lead to subtle differences in reported values [87,88,89,90,91,92]. This highlights the fact that the results obtained using different aberrometry methods are not directly comparable.

After cataract surgery with IOL implantation, vertical, horizontal coma, and spherical aberration tended to be higher than in phakic eyes, reflecting the loss of the natural lens’ compensatory effect on corneal aberrations. In pseudophakic eyes, corneal wavefront aberrations more closely represent the overall optical quality. However, the lack of correlation for certain terms underscores the limitations of corneal topography alone and the need for both corneal and total wavefront measurements for a comprehensive postoperative assessment [93].

Computer-based models can accurately predict postoperative aberrations after cataract surgery and correlate well with data measured in vivo. In the future, such individualized optical modeling might largely optimize IOL selection and surgical planning, optimizing patient-specific outcomes [94].

From a clinical perspective, the evaluation of HOAs may support a more individualized approach to cataract surgery, especially regarding IOL selection in patients with high visual demands, considering premium implants. Preoperative assessment of the patient’s corneal HOAs, ocular surface, and pupil size may assist in selecting the IOL design that better compensates for every individual’s optical profile. Additionally, minimizing the surgical-induced aberrations by careful incision planning and precise IOL positioning contribute to improved postoperative outcomes.

This review has several limitations. The available evidence is characterized by substantial heterogeneity regarding study designs, patient populations, and outcome measures. Variability in measurement techniques and devices limits direct comparisons across studies. Many studies applied specific selection strategies based on ocular surface status, corneal regularity, pupil size, visual demands, and patient expectations. Moreover, differences in surgical techniques, IOL design, and reporting standards may have influenced reported outcomes, potentially contributing to variability across studies and limiting direct comparisons. The implementation of a standardized framework regarding preoperative selection algorithms and aberrometric evaluation protocols would allow for more robust comparison between outcomes of different IOL designs and surgical techniques. Variability was also observed at a methodological level, as study designs ranged from randomized controlled trials to retrospective observational analyses, with considerable variability in sample size and follow-up duration. Taken together, this multidimensional heterogeneity explains part of the variability in reported outcomes. Potential publication bias cannot be excluded.

## 5. Conclusions

This scoping review aims to highlight the importance of high-order aberrations in the context of cataract surgery. Although not the only factor, they significantly influence the final postoperative visual quality. The evidence indicates that surgical techniques, intraocular lens design, corneal characteristics, measurement methodology, and patient-specific factors such as tear-film instability and pupil size all contribute to the final visual performance. While IOL design and surgical precision have reduced the HOA-related visual disturbances, heterogeneity persists across studies. Emerging lens concepts, such as enhanced monofocal designs, attempt to balance functional vision, further reflecting the shift toward individualized visual optimization in modern cataract surgery. Future research should focus on standardized wavefront measurement protocols, personalized surgical techniques, and IOL selection strategies, incorporating patient-specific characteristics in order to optimize postoperative outcomes.

## Figures and Tables

**Table 1:** Eligibility criteria used to identify relevant studies for inclusion in this scoping review. Studies were eligible if they involved patients undergoing cataract surgery and reported the measurement of higher-order aberrations (HOAs). All cataract surgical techniques and intraocular lens types were considered. A broad range of studies was included to map the existing evidence comprehensively, while non-clinical studies, animal studies, and articles lacking HOA measurement were excluded.

Criterion	Inclusion	Exclusion
Population	Patients undergoing cataract surgery	Animal studies
Concept	High-order aberrations	Studies Without high-order aberration measurement
Context	Any cataract surgery techniques	Non-cataract ocular surgery
Study types	Randomized controlled trials, cohort studies, cross-sectional, case series, case–control, systematic or narrative reviews	Editorials, letters
Language	English	Non-English

## Data Availability

No data were generated in this scoping review. All data supporting the findings of this study are derived from published articles cited in the reference list.

## Abbreviations

The following abbreviations are used in this manuscript:

HOA	higher-order aberration
IOL	intraocular lens
EDOF	extended depth-of-focus
FLACS	femtosecond-assisted cataract surgery
RMS	root mean square
CCI	clear corneal incision
